# Endometrial BCL6 Expression and Reproductive Outcomes in Infertile Women: A Systematic Review

**DOI:** 10.3390/diagnostics16111714

**Published:** 2026-06-02

**Authors:** Ana Maria Mihoci, Demetra Socolov, Olga Odetta Duma, Ruxandra Daniela Dumitrescu, Eduard Cristian Mihoci, Irina Voicu, Andreea Ioana Pruteanu, Bogdan Toma, Răzvan Vladimir Socolov

**Affiliations:** 1 Grigore T. Popa University of Medicine and Pharmacy, 16 University Street, 700115 Iasi, Romania; demetra.socolov@umfiasi.ro (D.S.); olga.duma@umfiasi.ro (O.O.D.); mihoci.eduard-cristian@d.umfiasi.ro (E.C.M.); andreea-ioana.pruteanu@umfiasi.ro (A.I.P.); razvan.socolov@umfiasi.ro (R.V.S.); 2 Clinical Hospital of Obstetrics and Gynecology “Elena Doamna”, 700038 Iasi, Romania; 3 Clinical Hospital of Obstetrics and Gynecology “Cuza Voda”, 700038 Iasi, Romania; 4 Department of Mother and Child Medicine, Grigore T. Popa University of Medicine and Pharmacy, 700115 Iasi, Romania; 5 Clinica Fertilia, 111 Popa Nan Street, 030581 Bucharest, Romania; ruxandra.dumitrescu@fertilia.ro (R.D.D.); irina.voicu@fertilia.ro (I.V.); 6 Department of Morpho-Functional Sciences I, Grigore T. Popa University of Medicine and Pharmacy, 700115 Iasi, Romania

**Keywords:** BCL6, endometrium, infertility, endometriosis, embryo transfer, implantation, clinical pregnancy, live birth, biomarker, reproductive outcomes

## Abstract

**Background/Objectives:** Endometrial B-cell lymphoma 6 (BCL6) overexpression has been proposed as a marker of progesterone resistance, occult endometriosis, and endometrial dysfunction in infertile women. Its possible association with reproductive outcomes and its potential role in guiding management before embryo transfer have attracted increasing interest. However, the prognostic and clinical significance of BCL6 across infertility settings remains incompletely defined. We systematically reviewed the literature on endometrial BCL6 expression and reproductive outcomes in infertile women. **Methods:** This systematic review was conducted according to a prospectively registered PROSPERO protocol. Studies evaluating endometrial BCL6 expression on biopsy in infertile women were eligible if they reported reproductive outcomes or clinically relevant comparative data. Two prespecified questions were addressed: whether high or abnormal BCL6 expression is associated with poorer reproductive outcomes than low or normal expression, and whether treatment before embryo transfer improves outcomes in BCL6-positive women. Thirteen studies were included in the review. Because the included studies differed substantially in infertility phenotype, embryo context, hormonal preparation, biopsy timing, and outcome reporting, findings were synthesized narratively. **Results:** The available evidence suggests that abnormal or elevated endometrial BCL6 expression may be associated with poorer reproductive outcomes in some infertility populations, particularly in women with unexplained infertility undergoing IVF, although this pattern was less evident in selected euploid embryo transfer cohorts. Treatment-oriented studies suggested a possible benefit of pre-transfer medical suppression or surgical management in some BCL6-positive women, but findings were variable across studies. Supportive non-comparative and mechanistic studies further indicated that interpretation of BCL6 is influenced by hormonal preparation, endometrial context, and whether BCL6 is considered in isolation or within a broader biological or multimarker framework. Interpretation of the available evidence was constrained by the limited number of comparative studies, observational study designs, variability in clinical populations, and incomplete standardization of reproductive outcome reporting. **Conclusions:** Endometrial BCL6 appears biologically and clinically relevant as a marker of endometrial dysfunction in selected infertility settings, but current evidence is insufficient to support routine stand-alone clinical use or strong treatment recommendations. Its apparent prognostic and clinical utility varies across patient populations and reproductive settings. Larger prospective comparative studies with harmonized biopsy protocols, standardized outcome reporting, and independent external validation are needed to clarify the role of BCL6 in reproductive medicine.

## 1. Introduction

Infertility remains a major clinical challenge, and despite substantial advances in assisted reproductive technologies (ART), implantation failure and suboptimal reproductive outcomes continue to affect a considerable proportion of patients [1,2,3,4]. Even in contemporary practice, embryo-related factors do not fully explain unsuccessful cycles, particularly in women with unexplained infertility, recurrent implantation failure, or recurrent pregnancy loss [1,4,5,6,7,8,9]. This has intensified interest in the endometrium as an active determinant of reproductive success rather than a passive substrate for embryo transfer [1,2,3,4,10]. Endometrial receptivity is essential for implantation, ongoing pregnancy, and live birth, and impaired endometrial function is increasingly recognized as an important contributor to reproductive failure [1,2,3,4,10].

Among the biomarkers proposed to reflect this dimension of endometrial dysfunction, B-cell lymphoma 6 (BCL6) has attracted growing interest [11,12,13,14]. BCL6 is a transcriptional repressor linked to progesterone resistance, inflammatory signaling, and endometriosis-associated endometrial dysfunction [11,12,13]. Increased endometrial BCL6 expression has been reported in women with endometriosis and unexplained infertility, and an HSCORE threshold of 1.4 has been widely adopted in subsequent studies evaluating abnormal expression [12,13,15]. Because endometriosis remains underrecognized in infertility care, BCL6 has also been proposed as a surrogate marker of occult endometriosis or related inflammatory pelvic pathology [12,14,16]. This interpretation is supported by reports of high positive predictive value of abnormal BCL6 expression for surgically identified endometriosis or other inflammatory pelvic abnormalities in selected infertility cohorts [16].

Despite increasing clinical interest, the clinical interpretation of endometrial BCL6 remains variable across infertility settings. Some studies have reported an association between abnormal BCL6 expression and poorer reproductive outcomes, particularly in women with unexplained infertility undergoing IVF [15]. Almquist et al. [15] observed substantially lower clinical pregnancy and live birth rates among women with elevated BCL6 expression.

However, studies conducted in more selected euploid embryo transfer populations did not reproduce the same pattern consistently. Klimczak et al. [17] found no significant association between BCL6 positivity and live birth after single euploid embryo transfer, while Strug et al. [18] similarly reported no clear relationship between BCL6 levels and subsequent euploid frozen embryo transfer outcomes. Together, these findings suggest that the prognostic relevance of BCL6 may vary according to clinical context, embryo selection strategy, biopsy conditions, and hormonal environment rather than representing a uniform marker across infertility populations.

Uncertainty also extends to the proposed clinical utility of BCL6. Treatment-oriented studies have suggested that medical suppression or laparoscopic management before embryo transfer may improve outcomes in BCL6-positive women, supporting the hypothesis that BCL6 identifies a potentially modifiable inflammatory or endometriosis-related endometrial phenotype [19,20]. In the prospective cohort reported by Likes et al. [19], women with abnormal BCL6 expression who underwent treatment before embryo transfer had substantially better implantation, clinical pregnancy, and live birth outcomes than untreated controls. However, this signal has not been reproduced consistently. Taggar et al. did not demonstrate significant differences in implantation, miscarriage, or ongoing pregnancy between treated and untreated BCL6-positive women, raising concern that a single biomarker may not reliably support treatment decisions in infertility care [20].

Interpretation of BCL6 is also influenced by methodological and biological variability. Measured BCL6 expression appears to vary according to uterine preparation protocol, with lower levels observed in progesterone-exposed endometrium than in natural-cycle samples, raising concerns regarding cross-study comparability [21]. In addition, BCL6 positivity does not align consistently with all endometrial receptivity testing frameworks. Huang et al. [22] reported discordance between positive ReceptivaDx BCL6 results and ERA-defined prereceptivity, suggesting that BCL6 may reflect a more specific inflammatory or progesterone-resistance-associated endometrial phenotype rather than a universal defect in receptivity. Together, these findings support the biological relevance of BCL6 while also highlighting the need for cautious clinical interpretation.

Against this background, a focused synthesis of the literature is warranted. Although endometrial BCL6 testing is increasingly discussed in infertility care, the available evidence has not been synthesized clearly in a manner that distinguishes prognostic association from treatment-related clinical utility [2,10,23,24]. We therefore conducted a systematic review to evaluate the relationship between endometrial BCL6 expression and reproductive outcomes in infertile women, as well as the potential clinical relevance of treatment before embryo transfer in BCL6-positive women.

Specifically, we addressed two prespecified clinical questions: first, whether high or abnormal endometrial BCL6 expression is associated with poorer reproductive outcomes than low or normal expression; and second, whether treatment before embryo transfer improves reproductive outcomes among BCL6-positive women compared with no treatment. A particular strength of the present review is the explicit separation of prognostic association from treatment-related clinical utility, two concepts that require distinct clinical interpretation.

## 2. Materials and Methods

### 2.1. Protocol and Registration

This systematic review was conducted according to a prospectively registered protocol in the International Prospective Register of Systematic Reviews (PROSPERO; CRD420261359172; registered on 3 April 2026). The review was designed to evaluate the association between endometrial BCL6 expression and reproductive outcomes in infertile women, as well as the potential clinical relevance of treatment before embryo transfer in BCL6-positive women. It was structured around two prespecified clinical questions: (1) whether high or abnormal endometrial BCL6 expression is associated with poorer reproductive outcomes than low or normal expression; and (2) whether treatment before embryo transfer improves reproductive outcomes among BCL6-positive women compared with no treatment. Review conduct and reporting were aligned with PRISMA 2020 principles for systematic reviews [25].

Although meta-analysis was initially considered at the protocol stage if feasible, the final evidence base proved too sparse and clinically heterogeneous to support a robust formal quantitative synthesis. Accordingly, the review was completed and is reported as a systematic review with structured narrative synthesis. This represents a transparent methodological refinement based on the characteristics of the available evidence rather than a post hoc attempt to alter the review question.

### 2.2. Eligibility Criteria

Eligibility criteria were defined a priori in accordance with the review objectives.

#### 2.2.1. Population

Eligible studies enrolled women of reproductive age undergoing evaluation or treatment for infertility, including unexplained infertility, recurrent implantation failure, recurrent pregnancy loss, and IVF or frozen embryo transfer populations, in whom endometrial BCL6 expression had been assessed on endometrial biopsy. For the treatment question, studies of BCL6-positive women undergoing pre-transfer intervention were eligible when an untreated, no-treatment, or usual-care comparator was available.

Studies were excluded if they enrolled animal or in vitro models, male participants, adolescent populations, or women without infertility-related reproductive outcomes. Studies evaluating BCL6 only as a diagnostic marker without clinical reproductive outcomes, studies without biopsy-based endometrial BCL6 assessment, and studies without sufficient clinically relevant comparative data were also excluded.

#### 2.2.2. Exposure and Intervention

For the prognostic question, the exposure of interest was endometrial BCL6 expression assessed on biopsy and categorized as high/abnormal versus low/normal, or as positive versus negative according to a clearly reported study-specific threshold, most commonly an HSCORE cut-off of 1.4. For the treatment question, eligible interventions included pre-transfer medical suppression and/or surgical management undertaken in BCL6-positive women.

Studies were excluded if BCL6 was not assessed in endometrial tissue, if a relevant comparator structure was absent, or if reproductive outcomes were not reported.

#### 2.2.3. Comparators

For prognostic analyses, the comparator was low or normal endometrial BCL6 expression. For treatment analyses, the comparator was no treatment, usual care, or untreated controls among BCL6-positive women. Studies without an appropriate comparator group were not considered suitable for the main comparative interpretation.

#### 2.2.4. Outcomes

The primary outcomes were clinical pregnancy rate and live birth rate. Secondary outcomes included implantation rate, ongoing pregnancy or delivery, miscarriage rate, and biochemical pregnancy rate, where reported. Outcomes were evaluated separately for each prespecified clinical question and interpreted in relation to study design, clinical context, and reporting structure.

#### 2.2.5. Study Designs

Eligible designs included randomized studies and non-randomized studies, including prospective and retrospective cohort studies, case–control studies, and other observational designs relevant to the review questions. Comparative studies reporting reproductive outcomes according to endometrial BCL6 status or treatment status among BCL6-positive women were prioritized for the main synthesis. Additional non-randomized observational studies considered clinically informative but not fully aligned with the main comparative framework were eligible for narrative synthesis.

Narrative reviews, systematic reviews, editorials, letters, case reports, very small case series, and conference abstracts without sufficient extractable or interpretable data were excluded from the primary evidence synthesis. Abstract-only evidence considered clinically informative but not fully eligible under the prespecified framework was retained only as contextual narrative support.

### 2.3. Information Sources and Search Strategy

A systematic literature search was undertaken across major biomedical databases, including MEDLINE/PubMed, Embase, CENTRAL, Science Citation Index, and Scopus, with supplementary searching of reference lists and citation chains. Searches were conducted between 15 December 2025 and 1 March 2026, with database coverage extending from inception to 1 March 2026. Detailed database-specific electronic search strategies, including search period, database coverage, source-specific syntax, and additional identification methods, are provided in Supplementary Table S1 [26].

The search strategy combined controlled vocabulary terms, including MeSH and database-specific indexed terms where applicable, with free-text keywords related to infertility, endometrial biopsy, BCL6, embryo transfer, implantation, pregnancy outcomes, recurrent implantation failure, recurrent pregnancy loss, and assisted reproduction. No language or date restrictions were applied. Additional identification methods included reference list screening of included studies and relevant reviews, forward citation searching, and contact with authors or experts where required.

### 2.4. Screening and Eligibility Assessment

Study selection was performed in two stages. Titles and abstracts were screened first, followed by full-text assessment of potentially relevant reports against the predefined eligibility criteria. Screening was conducted independently by two reviewers, with disagreements resolved by discussion and, when necessary, consultation with a third reviewer, in accordance with the registered protocol.

**Figure 1:**
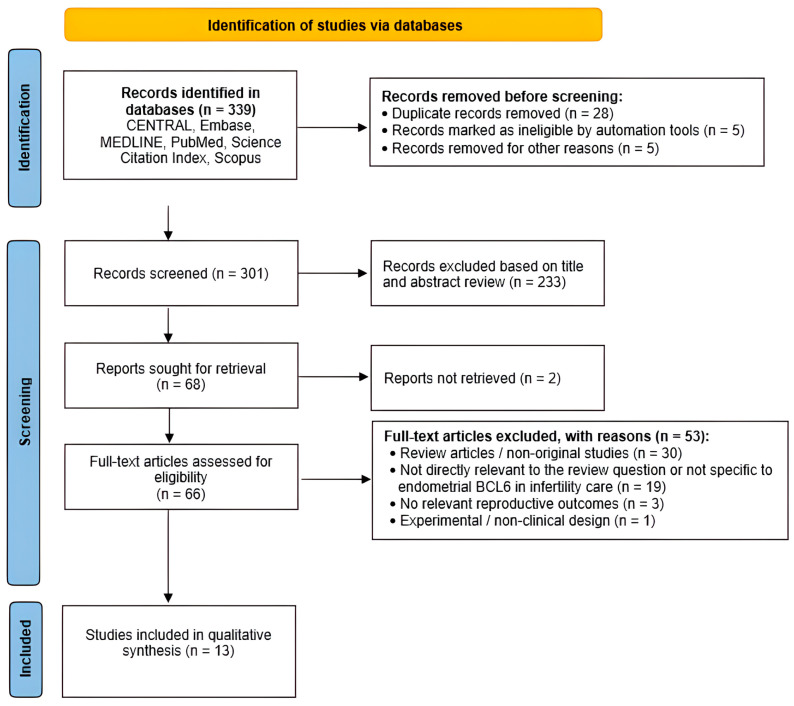
PRISMA 2020 flow diagram of study selection.

The selection process is summarized in the PRISMA 2020 flow diagram (Figure 1).

### 2.5. Data Extraction

Data extraction was performed independently by two reviewers using a predefined framework designed to maximize methodological comparability and clinical interpretability. Disagreements were resolved by discussion and, where required, by consultation with a third reviewer, consistent with the registered protocol.

Extracted variables included study author, publication year, country, study design, infertility phenotype, ART setting, sample size, embryo context, timing of endometrial biopsy, BCL6 assessment method, threshold for abnormality, comparator structure, intervention details for treatment studies, and reported reproductive outcomes. Where available, comparative outcome data were extracted for clinical pregnancy, live birth, implantation, ongoing pregnancy or delivery, and miscarriage. Studies considered clinically informative but not providing directly interpretable comparative reproductive outcome data in a form aligned with the main review questions were retained for narrative synthesis only.

### 2.6. Risk-of-Bias Assessment

Risk of bias was assessed according to study design and analytical role in the review. For non-randomized intervention studies comparing treated versus untreated BCL6-positive women, a ROBINS-I-informed domain-based approach was used, covering confounding, participant selection, classification of interventions, deviations from intended interventions, missing data, outcome measurement, and selective reporting [27]. For prognostic comparative studies evaluating high/abnormal versus low/normal BCL6 expression, assessment focused on study participation, comparator definition, exposure measurement, confounding, outcome assessment, missing data, and statistical reporting [28].

Because the evidence base consisted predominantly of observational studies conducted in clinically selected populations, particular attention was paid to confounding, comparator structure, and indirectness. Overall, the main comparative evidence was judged to range from moderate to serious risk of bias, with confounding by indication representing the principal limitation in treatment studies. Risk-of-bias assessment was performed independently by two reviewers using the prespecified domain-based approach, with disagreements resolved by discussion and, where necessary, consultation with a third reviewer.

### 2.7. Data Synthesis

All included studies were synthesized narratively, and narrative synthesis constituted the primary method of evidence synthesis. The two review questions were considered separately: (1) high/abnormal versus low/normal endometrial BCL6 expression; and (2) treated versus untreated BCL6-positive women before embryo transfer.

Findings were organized according to comparison, outcome, infertility phenotype, ART setting, embryo context, timing and hormonal context of endometrial biopsy, BCL6 assessment method, comparator structure, and intervention strategy.

Particular attention was paid to direction of effect, the consistency of findings across clinically comparable studies, and the extent to which differences in infertility phenotype, embryo-transfer setting, hormonal preparation, biopsy timing, and ART context appeared to influence interpretation.

Formal quantitative pooling was not undertaken because the comparative evidence was limited and clinically diverse, with substantial variability in infertility phenotype, embryo-transfer context, BCL6 assessment strategy, comparator structure, and reproductive outcome reporting.

In addition, the number of directly comparable studies for each outcome was too small to support a robust and clinically meaningful quantitative summary. Accordingly, the evidence was interpreted through structured narrative comparison rather than formal meta-analysis.

To improve interpretability and assess the robustness of the narrative findings, sensitivity-oriented interpretation was applied by examining whether observed associations remained directionally similar across studies with comparable clinical characteristics and whether conclusions differed according to study design or ART context.

### 2.8. Certainty and Interpretive Framework

Because the included evidence was sparse, predominantly observational, and clinically variable, overall certainty was interpreted cautiously. Particular attention was paid to risk of bias, inconsistency, indirectness, and imprecision when formulating the review conclusions. A structured overview of the principal sources of heterogeneity across included studies is provided in Supplementary Table S2, a structured narrative summary of the direction, consistency, and interpretability of the evidence across the prespecified clinical questions is provided in Supplementary Table S3, and the certainty of evidence for the main reproductive outcomes was assessed using the GRADE approach and is presented in Supplementary Table S4 [29].

### 2.9. Ethical Considerations

As this study was based exclusively on published literature and did not involve direct collection of individual patient data, ethical approval and informed consent were not required.

## 3. Results

### 3.1. Study Selection

The study selection process is shown in Figure 1. A total of 339 records were identified through database searching across CENTRAL, Embase, MEDLINE, PubMed, Science Citation Index, and Scopus. After removal of 28 duplicates, 5 records excluded by automation tools, and 5 records removed for other reasons, 301 records underwent title and abstract screening. Of these, 233 were excluded, leaving 68 reports sought for retrieval; 2 could not be retrieved. Sixty-six full-text reports were assessed for eligibility, of which 53 were excluded, primarily because they were review or other non-original articles, were not directly relevant to the review question, did not report eligible reproductive outcomes, or used an experimental or otherwise non-clinical design. Thirteen studies were included in the qualitative synthesis. Detailed reasons for full-text exclusion are provided in Supplementary Table S5.

A conference abstract by Angress et al. [30] was identified during screening and was directionally consistent with the published treatment-oriented literature; however, it was not retained in the formal evidence synthesis because it did not meet the prespecified PROSPERO eligibility framework. Specifically, it was available only in abstract form, used a comparator structure not fully aligned with the treatment question, and reported a composite live birth/ongoing pregnancy endpoint rather than a directly interpretable predefined outcome. It was therefore retained only as supportive contextual evidence [30].

### 3.2. Characteristics of Included Studies

The principal characteristics of the included studies are summarized in Table 1. Thirteen studies published between 2016 and 2025 were included in the review. These comprised prospective and retrospective cohort studies, retrospective chart reviews, case–control studies, and methodological or mechanistic observational studies. The included populations were clinically heterogeneous and encompassed women with unexplained infertility, recurrent implantation failure, recurrent pregnancy loss, suspected or confirmed endometriosis, and assisted reproduction populations undergoing IVF, embryo transfer, or frozen embryo transfer [12,13,15,16,17,18,19,20,21,22,31,32,33].

Endometrial BCL6 expression was generally assessed on biopsy by immunohistochemistry, most commonly using HSCORE-based definitions of abnormality. However, substantial between-study variation was present with respect to biopsy timing, hormonal context, comparator structure, embryo context, and reported reproductive outcomes. These differences were central to interpretation of both the prognostic and treatment-oriented evidence [12,13,15,16,17,18,19,20,21,22,31,32,33].

Three comparative studies addressed the prognostic association between high/abnormal versus low/normal endometrial BCL6 expression and reproductive outcomes. Almquist et al. [15] evaluated women with unexplained infertility undergoing IVF and reported clinical pregnancy and live birth according to BCL6 status. Klimczak et al. [17] assessed BCL6 in a normal-responder IVF population undergoing single euploid embryo transfer and reported live birth. Strug et al. [18] prospectively evaluated BCL6/SIRT1 levels in a fresh-cycle biopsy setting and examined their relationship with subsequent euploid frozen embryo transfer outcomes.

Two comparative studies addressed treatment before embryo transfer in BCL6-positive women. Likes et al. [19] compared pre-transfer medical suppression or laparoscopy with no treatment in women with abnormal BCL6 expression [19]. Taggar et al. reported implantation, miscarriage, and ongoing pregnancy/delivery outcomes in treated and untreated BCL6-positive women within a retrospective infertility cohort [20].

The remaining eight studies were retained for contextual narrative interpretation because they were clinically informative but did not provide a comparator structure fully aligned with the main comparative questions or did not report directly interpretable comparative reproductive outcome data. These studies included biological, diagnostic, methodological, mechanistic, and multimarker investigations that were important for broader interpretation of BCL6 but were not central to the main comparative synthesis [12,13,16,21,22,31,32,33]. The principal sources of clinical and methodological heterogeneity across included studies are summarized in Supplementary Table S2.

### 3.3. Risk of Bias

**Figure 2:**
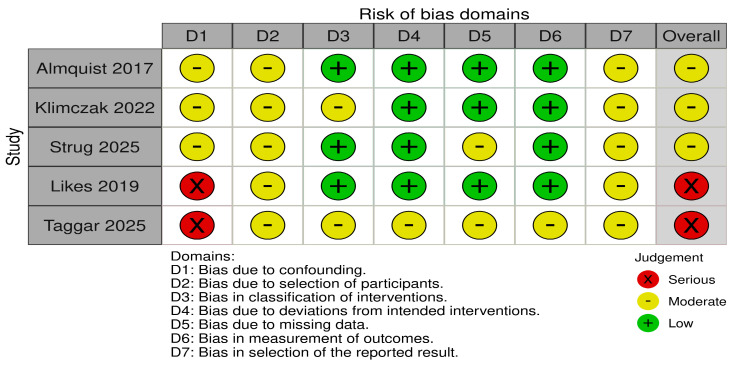
Study-level domain-based risk-of-bias assessment of the five comparative studies central to the review questions [15,17,18,19,20].

**Figure 3:**
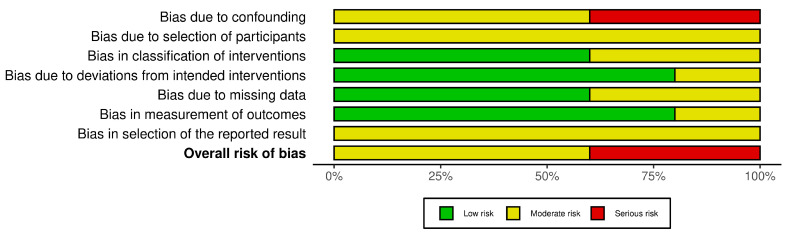
Weighted summary of domain-based risk-of-bias judgments across the five comparative studies central to the review questions [15,17,18,19,20].

The domain-based risk-of-bias assessment for the five comparative studies central to the review questions is presented in Figure 2, and the weighted distribution of judgments across domains is shown in Figure 3. Study-level judgments for all included studies are summarized in Table 2. Overall, the internal validity of the comparative evidence was limited by the observational design of the included studies, clinically selected infertility populations, and incomplete control for confounding [15,17,18,19,20,27,28].

At the study level, Almquist 2017 [15], Klimczak 2022 [17], and Strug 2025 [18] were judged to be at overall moderate risk of bias, whereas Likes 2019 [19] and Taggar 2025 [20] were judged to be at overall serious risk of bias. As shown in Figure 2, confounding was the dominant source of bias across studies. It was rated as serious in the two treatment-oriented studies and moderate in the three prognostic studies, reflecting the non-randomized nature of the intervention comparisons and the likelihood that treatment allocation was influenced by underlying clinical severity, reproductive history, or physician decision-making [19,20,27,28].

Selection bias was judged as moderate across the five comparative studies, reflecting clinically selected infertility populations and the limited generalizability inherent to highly specific reproductive medicine cohorts. Exposure/intervention assessment and outcome assessment were generally judged to be at low to moderate risk, although moderate concern remained in some treatment comparisons because of retrospective ascertainment and heterogeneous intervention pathways. Missing data were judged predominantly as low to moderate risk. Selective reporting was consistently judged as moderate, largely because of incomplete standardization of reproductive outcome reporting and limited analytical prespecification [15,17,18,19,20,27,28].

The weighted summary shown in Figure 3 reinforces this overall pattern. Confounding was the only domain contributing serious risk at the aggregate level, whereas selection bias and selective reporting were dominated by moderate-risk judgments. By contrast, exposure/intervention assessment, outcome assessment, and missing data were more often judged at low risk, although not uniformly so. Taken together, this risk-of-bias profile supports cautious interpretation of the comparative findings, particularly for the treatment question, for which confounding by indication represents the principal methodological concern [19,20,27,28].

### 3.4. Findings for Comparison 1: High/Abnormal Versus Low/Normal Endometrial BCL6 Expression

The comparative findings for the prognostic question are summarized in Table 3. Three studies informed this comparison. Overall, the available evidence suggested that high or abnormal endometrial BCL6 expression may be associated with poorer reproductive outcomes in some infertility populations, but this association was not reproduced uniformly across clinical settings [15,17,18].

In Almquist 2017 [15], women with low/normal BCL6 expression had substantially better reproductive outcomes than women with high/abnormal expression. Clinical pregnancy occurred in 11/17 (64.7%) women with low/normal BCL6 compared with 9/52 (17.3%) women with high/abnormal BCL6, and live birth occurred in 10/17 (58.8%) versus 6/52 (11.5%), respectively. These findings support an adverse prognostic association of BCL6 overexpression in women with unexplained infertility undergoing IVF before embryo transfer [15].

By contrast, Klimczak 2022 [17] did not identify a significant association between BCL6 positivity and live birth in a highly selected normal-responder IVF population undergoing warmed single euploid embryo transfer. As shown in Table 3, live birth occurred in 20/35 (57.1%) women in the BCL6-negative group and 7/15 (46.7%) women in the BCL6-positive group, with no statistically significant difference between groups [17].

Similarly, Strug 2025 [18] did not demonstrate a clear association between BCL6 or SIRT1 levels and subsequent pregnancy outcomes after euploid frozen embryo transfer. Although the study was clinically informative for narrative interpretation, it did not provide directly interpretable comparative event counts in a simple low-versus-high BCL6 format aligned with the main comparative question [18].

Taken together, these findings suggest that any apparent prognostic value of endometrial BCL6 is likely to be context-dependent. The strongest adverse signal was observed in women with unexplained infertility undergoing IVF without strict euploid embryo control, whereas more selected euploid-transfer cohorts did not show a comparable signal [15,17,18].

### 3.5. Findings for Comparison 2: Treated Versus Untreated BCL6-Positive Women Before Embryo Transfer

The comparative findings for the treatment question are summarized in Table 4. Two studies directly addressed whether treatment before embryo transfer improved reproductive outcomes among BCL6-positive women. Overall, the available evidence suggested a possible treatment benefit in selected settings, but the magnitude and consistency of the observed effect differed substantially across studies [19,20].

In Likes 2019 [19], women with abnormal BCL6 expression who underwent medical suppression or laparoscopy before embryo transfer had better reproductive outcomes than untreated controls. As summarized in Table 4, implantation rates were 9/21 (42.9%) after GnRH agonist treatment and 18/45 (40.0%) after laparoscopy, compared with 12/103 (11.7%) in untreated women. Clinical pregnancy occurred in 6/10 (60.0%) and 13/21 (61.9%) of treated women, respectively, compared with 8/54 (14.8%) in untreated controls. For live birth, the combined treated groups accounted for 16/31 (51.6%) live births compared with 4/54 (7.4%) in untreated women. Miscarriage was also less frequent in treated women, although the number of events was small and precision was limited. These findings provide the strongest direct comparative signal in favor of pre-transfer treatment among BCL6-positive women [19].

By contrast, Taggar 2025 [20] did not confirm a clear treatment effect. As shown in Table 4, implantation occurred in 11/17 (65%) treated women and 3/5 (60%) untreated women, ongoing pregnancy occurred in 9/17 (53%) and 2/5 (40%), respectively, and miscarriage occurred in 2/17 (12%) and 1/5 (20%). No statistically significant between-group differences were reported. Although the sample size was small and the study was underpowered for definitive comparative inference, these findings did not reproduce the stronger treatment signal observed in Likes 2019 [19].

Taken together, the treatment-oriented literature suggests that pre-transfer intervention in BCL6-positive women may improve reproductive outcomes in some clinical contexts, but the current evidence remains limited and methodologically vulnerable. Differences in study design, infertility phenotype, intervention strategy, and risk of confounding support cautious interpretation of the apparent treatment signal [19,20,27].

### 3.6. Narrative-Only Supportive Studies

Several additional studies were not central to the main comparative questions but were important for interpreting the broader biological, methodological, and clinical relevance of endometrial BCL6. These studies are summarized in Table 1, while their contribution to clinical and methodological heterogeneity and to the overall interpretive framework is further synthesized in Supplementary Tables S2 and S3 [12,13,16,21,22,31,32,33].

Collectively, these studies help explain why the comparative evidence remains difficult to interpret across infertility settings. Fox 2019 [13] showed that endometrial BCL6 expression was elevated in women with unexplained infertility and unexplained recurrent pregnancy loss compared with fertile controls, extending the relevance of this marker beyond a narrowly defined IVF-only setting [13]. Huang 2023 [21] demonstrated that measured BCL6 expression differed substantially according to uterine preparation protocol, with lower levels observed in progesterone-exposed endometrium than in natural-cycle samples, indicating that hormonal context at the time of biopsy may materially influence test interpretation [21]. Huang 2025 [22] further reported discordance between a positive ReceptivaDx BCL6 result and ERA-defined prereceptivity, suggesting that BCL6 should not be interpreted as a universal stand-alone marker of endometrial receptivity failure, but rather as a more specific inflammatory or progesterone-resistance-associated endometrial phenotype [22].

Other supportive studies were informative from mechanistic and multimarker perspectives. Lessey 2024 [33] suggested that suppression of suspected endometriosis/BCL6-related pathology may modify inflammatory and epigenetic pathways in women with unexplained euploid embryo transfer failure [33]. Ekemen 2023 [31] evaluated BCL6 within a broader immunohistochemical panel that also included CD56 and CD138, supporting the view that BCL6 may be more informative when interpreted within a multimarker framework rather than in isolation [31].

Foundational and methodological studies further reinforced the broader interpretive context. Evans-Hoeker 2016 [12] supported the association between elevated endometrial BCL6 and endometriosis/unexplained infertility and contributed to the widely used HSCORE threshold of 1.4. Nezhat 2020 [16] reported high positive predictive value of positive endometrial BCL6 for endometriosis or other inflammatory pelvic pathology in a selected IVF population. Squatrito 2022 [32] highlighted the methodological importance of how BCL6 is quantified, showing that digital-assisted analysis correlated with conventional HSCORE while potentially improving objectivity and reproducibility.

These supportive studies were not central to the main comparative synthesis because they lacked directly aligned comparator structures, focused primarily on mechanistic or methodological questions, or did not report reproductive outcome data in a form directly aligned with the prespecified review questions. Nevertheless, they remain important for interpreting the broader clinical meaning of BCL6 and for understanding why findings may differ across study designs and patient populations [12,13,16,21,22,31,32,33].

### 3.7. Overall Synthesis of Results

Taken together, the available evidence suggests that endometrial BCL6 expression may have both prognostic and clinical relevance in infertility care; however, the current literature remains sparse, variable, and methodologically constrained. Across the comparative prognostic studies summarized in Table 3, high or abnormal BCL6 expression was associated with poorer reproductive outcomes in some infertility populations, particularly in women with unexplained infertility undergoing IVF, but this association was not reproduced consistently across all clinical settings and was less evident in selected euploid embryo transfer cohorts. Across the treatment-oriented studies summarized in Table 4, pre-transfer intervention in BCL6-positive women appeared beneficial in one study but not in another, leaving the clinical actionability of a positive BCL6 result incompletely defined [15,17,18,19,20].

Viewed together, the comparative evidence does not support a simple or uniform interpretation of BCL6 across infertility populations. Rather, it suggests that the apparent relevance of BCL6 is strongly clinically variable. The adverse prognostic signal was most evident in broader unexplained infertility settings without strict euploid embryo control, whereas studies conducted in more selected euploid transfer populations did not demonstrate the same pattern. Similarly, the treatment-oriented evidence suggested possible benefit in some BCL6-positive women, but this signal was variably reproduced across studies and remained vulnerable to confounding by indication, differences in intervention strategy, and limited statistical power [15,17,18,19,20,27,28].

This uncertainty is reinforced by the broader contextual literature summarized in Table 1 and Supplementary Table S3. Several supportive studies suggest that BCL6 should not be interpreted as a universal stand-alone marker of receptivity failure, but rather as a marker of a more specific inflammatory or progesterone-resistance-associated endometrial phenotype. In this respect, the wider literature helps explain why findings differ across studies. The interpretation of BCL6 appears to be influenced by infertility phenotype, ART setting, embryo context, hormonal preparation, timing of biopsy, comparator structure, and whether BCL6 is considered in isolation or as part of a broader biological or multimarker framework [12,13,16,21,22,31,32,33].

The biological plausibility of BCL6 remains an important strength of the field. Endometrial BCL6 overexpression has been linked to progesterone resistance, inflammatory signaling, and endometriosis-associated endometrial dysfunction, and several of the included supportive studies reinforce the view that BCL6 reflects a clinically relevant dimension of endometrial abnormality. At the same time, biological plausibility alone does not establish stable clinical utility. The currently available studies differ substantially in design, patient selection, biopsy conditions, and outcome reporting, and these differences materially affect interpretation of the evidence [11,12,13,14,21,22].

Accordingly, the present review supports a cautious but clinically meaningful conclusion. Endometrial BCL6 appears to be a context-dependent marker of endometrial dysfunction that may be associated with adverse reproductive outcomes in selected infertility populations and may have treatment-responsive implications in some BCL6-positive women. However, the current literature remains insufficient to support routine stand-alone use of BCL6 as a definitive decision-guiding test across reproductive settings [15,17,18,19,20,21,22,27,28,29,31,32].

The overall certainty of evidence for the main reproductive outcomes, assessed using the GRADE approach, is summarized in Supplementary Table S4 [29]. Given the small number of comparative studies, the predominance of observational designs, the heterogeneity of infertility populations and ART settings, and the imprecision of reported outcome data, stronger clinical recommendations would be premature. Better-standardized prospective comparative studies with clearer clinical phenotyping, harmonized biopsy conditions, and consistently reported reproductive outcomes are needed before BCL6 can be considered a robust tool for routine reproductive practice [15,17,18,19,20,21,22,27,28,29,31,32].

## 4. Discussion

### 4.1. Principal Findings

This systematic review evaluated the prognostic and clinical relevance of endometrial BCL6 expression in infertile women across two prespecified clinical questions: first, whether high or abnormal endometrial BCL6 expression is associated with poorer reproductive outcomes than low or normal expression; and second, whether treatment before embryo transfer improves reproductive outcomes among BCL6-positive women [15,17,18,19,20].

The principal contribution of this review is not to support universal clinical adoption of BCL6 testing, but rather to clarify that the apparent clinical relevance of BCL6 varies across infertility settings. Overall, the available evidence suggests that abnormal endometrial BCL6 expression may be associated with poorer reproductive outcomes in selected infertility populations, whereas pre-transfer treatment in BCL6-positive women may improve outcomes in some clinical contexts. However, interpretation of the current evidence remains constrained by the limited number of comparative studies, predominantly observational study designs, variability in patient populations, and important methodological differences across studies [15,17,18,19,20,27,28,29].

For the prognostic comparison, the strongest adverse association was observed in Almquist 2017 [15], in which low or normal BCL6 expression was associated with substantially higher clinical pregnancy and live birth rates than high or abnormal expression in women with unexplained infertility undergoing IVF. By contrast, Klimczak 2022 [17] and Strug 2025 [18] did not identify a clear adverse association in more selected euploid embryo transfer settings. Together, these findings suggest that the prognostic relevance of BCL6 may vary across infertility populations and ART contexts [15,17,18].

For the treatment comparison, Likes 2019 [19] reported markedly better implantation, clinical pregnancy, and live birth outcomes after medical suppression or laparoscopy in BCL6-positive women, whereas Taggar 2025 [20] did not identify significant differences between treated and untreated groups. Interpretation of these findings remains limited by non-randomized allocation, small sample size, and substantial methodological differences between studies. Accordingly, the current evidence supports, at most, a potentially treatment-responsive signal in selected BCL6-positive populations rather than a broadly generalizable treatment effect [19,20].

### 4.2. Interpretation in Context

The present findings support the view that BCL6 is biologically and clinically relevant, but they do not support a simple or universal interpretation of a positive result. BCL6 has been linked to progesterone resistance, inflammatory signaling, and endometriosis-associated endometrial dysfunction, providing a plausible biological rationale for its association with impaired implantation and poorer reproductive outcomes [11,12,14]. Foundational studies showed increased endometrial BCL6 expression in women with endometriosis and unexplained infertility, and other work reported high positive predictive value of positive endometrial BCL6 for surgically identified endometriosis or inflammatory pelvic pathology in selected infertility populations [12,13,16]. These observations support the concept that BCL6 may identify an endometrial phenotype enriched for occult inflammatory or endometriosis-related dysfunction rather than serving as a generic marker of receptivity failure [11,12,13,14,16].

At the same time, the current review indicates that biological plausibility does not automatically translate into stable clinical performance across settings. One likely explanation for the inconsistency across studies is that BCL6 appears to be strongly not uniform across populations. The included literature differed substantially with respect to infertility phenotype, embryo ploidy control, timing of biopsy, uterine preparation protocol, and whether BCL6 was evaluated as an isolated biomarker or within a broader testing framework [15,17,18,19,20,21,22,31,32]. This is particularly relevant because Huang 2023 [21] showed that measured BCL6 expression varies according to uterine preparation method, with lower levels in progesterone-exposed endometrium than in natural-cycle samples, suggesting that hormonal exposure alone may materially influence test interpretation. Likewise, Huang 2025 [22] reported discordance between BCL6 positivity and ERA-defined prereceptivity, indicating that BCL6 should not be treated as a universal surrogate for all forms of endometrial receptivity failure.

Taken together, these findings suggest that BCL6 should be interpreted less as a universal receptivity marker and more as a context-sensitive indicator of a specific inflammatory or progesterone-resistance-associated endometrial phenotype [11,12,14,21,22]. This interpretation also helps explain why a strong adverse prognostic association was observed in broader unexplained infertility cohorts yet was not reproduced in selected euploid-transfer populations. In settings in which embryo-related variability is reduced, the residual contribution of BCL6-defined endometrial dysfunction may differ from that observed in less selected infertility populations. Similarly, the apparent treatment benefit reported in one study but not in another may reflect differences in underlying clinical phenotype, intervention selection, sample size, and confounding rather than a stable causal effect applicable to all BCL6-positive patients [15,17,18,19,20].

Supportive abstract-level evidence points in a similar general direction, although it should be interpreted cautiously. In a multicenter conference abstract, Angress 2020 [30] reported outcomes after ReceptivaDx-guided management across seven IVF centers, with numerically favorable outcomes among treated BCL6-positive women. However, this study was available only in abstract form, relied on self-reported multicenter data, used a comparator structure not fully aligned with the prespecified review question, and reported a composite live birth/ongoing pregnancy endpoint. It therefore provides supportive, but not confirmatory, evidence for possible clinical utility of treatment in selected BCL6-positive women [30].

### 4.3. Clinical Implications

From a clinical perspective, the present review does not support universal stand-alone use of endometrial BCL6 testing as a definitive decision tool in infertility care. The available evidence is insufficient to justify strong routine recommendations, either for prognosis or for treatment selection, across reproductive settings. Nevertheless, the findings do suggest that BCL6 may be clinically informative in selected contexts, particularly where occult endometriosis, progesterone resistance, recurrent implantation failure, or otherwise unexplained reproductive failure are suspected [11,12,13,14,15,16,17,18,19,20,21,22].

The most defensible interpretation at present is that BCL6 may contribute to individualized assessment rather than function as a binary rule-in or rule-out test. It is better viewed as a clinically variable biomarker whose meaning may vary according to patient selection, embryo testing strategy, biopsy timing, and endometrial preparation protocol. A positive result may indicate an endometrial environment more consistent with inflammatory or endometriosis-associated dysfunction, but its clinical significance appears to depend on the broader infertility phenotype, hormonal context, ART strategy, embryo selection approach, and comparator framework. The same caution applies to treatment-related interpretation. A major limitation affecting broader clinical implementation is the lack of standardization across studies, particularly regarding biopsy timing, hormonal preparation protocols, HSCORE interpretation, embryo-transfer context, and reproductive outcome reporting. Huang 2023 [21] further demonstrated that endometrial BCL6 expression may vary according to uterine preparation protocol, underscoring the need for assay harmonization and standardized testing frameworks before broader clinical implementation can be considered. Although some BCL6-positive women may benefit from pre-transfer intervention, the current evidence does not support assuming that treatment will improve outcomes uniformly across all patients with a positive test [15,17,18,19,20,21,22].

Accordingly, BCL6 is best viewed, at present, as a potentially informative component of a broader clinical framework rather than as a universally robust stand-alone biomarker. This interpretation is also supported by studies evaluating BCL6 within multimarker panels or in conjunction with mechanistic endometrial assessment rather than in isolation [22,31]. Another important limitation is that much of the comparative evidence originates from a relatively limited number of overlapping investigative groups. Although these studies are clinically informative and methodologically valuable, independent external validation across diverse infertility populations and clinical settings remains limited. In practical terms, a positive BCL6 result should be interpreted in conjunction with the overall infertility phenotype rather than used in isolation to direct treatment decisions.

### 4.4. Strengths and Limitations

A major strength of this review is the explicit separation of two clinically distinct questions: prognostic relevance and treatment-related clinical utility. This distinction avoided conflating biological association with clinical actionability and allowed a more coherent interpretation of the literature. Another strength is the integration of both supportive and contradictory findings across unexplained infertility, recurrent reproductive failure, and euploid embryo transfer settings. Structured narrative synthesis was considered appropriate given the limited number of directly comparable studies and the substantial clinical and methodological variability across the available literature [15,17,18,19,20,27,28,29].

Several limitations should be acknowledged. First, the number of eligible comparative studies was small. Second, most included evidence was observational, and the treatment comparison in particular remained vulnerable to confounding by indication. Third, the included studies differed substantially in patient population, embryo context, biopsy timing, hormonal exposure, BCL6 assessment strategy, comparator structure, and reported reproductive outcomes [15,17,18,19,20,21,22,27,28,29,30].

These limitations should be considered when interpreting the current evidence, particularly given the variability across infertility phenotypes, embryo-transfer settings, and treatment-selected populations. Accordingly, the present findings support cautious clinical interpretation and highlight the need for larger prospective comparative studies with standardized protocols and clinically meaningful reproductive outcomes [15,17,18,19,20,21,22,27,28,29].

### 4.5. Future Research

Future studies should prioritize prospective comparative designs with clearly defined infertility phenotypes, standardized biopsy timing, and harmonized BCL6 assessment methods. Reproductive outcomes should be reported consistently, particularly clinical pregnancy, live birth, implantation, ongoing pregnancy, and miscarriage, using transparent denominators and clearly described ART settings. Greater methodological consistency would improve both comparability across studies and interpretability of the evidence [15,17,18,19,20].

There is also a clear need for stronger studies evaluating treatment decisions based on BCL6 positivity. Ideally, such studies would compare specific interventions in well-characterized BCL6-positive populations and distinguish between medical suppression, surgical management, and combined strategies. Particular attention should be paid to potential effect modification by embryo ploidy control, hormonal preparation, and suspected endometriosis status. In parallel, future work should determine whether BCL6 has greater clinical value as an isolated biomarker or as part of a broader multimarker or phenotype-driven framework. Well-designed multicenter studies would be especially valuable in clarifying whether the more favorable signals observed in some cohorts persist under more rigorous prospective conditions [19,20,22,30,31].

## 5. Conclusions

Endometrial BCL6 expression appears biologically and clinically relevant as a marker of endometrial dysfunction and, in selected infertility populations, may be associated with poorer reproductive outcomes. The currently available evidence also suggests that pre-transfer treatment in BCL6-positive women may improve selected reproductive outcomes in some clinical settings. However, interpretation of the existing literature remains constrained by the limited number of comparative studies, predominantly observational study designs, variability in infertility populations and ART settings, and incomplete standardization of reproductive outcome reporting. Accordingly, BCL6 may currently be regarded as a potentially informative biomarker whose clinical interpretation depends on infertility phenotype, embryo context, hormonal environment, and biopsy conditions rather than as a universally applicable stand-alone tool for routine infertility decision-making. Current evidence supports individualized clinical interpretation rather than uniform test-directed management. Larger prospective comparative studies with standardized BCL6 assessment, clearly defined clinical phenotypes, and consistently reported reproductive outcomes are required before broader clinical implementation can be recommended [15,17,18,19,20,21,22,27,28,29].

## Figures and Tables

**Table 1:** General features of included studies.

Study	Country	Design	Population (*N*)	Clinical Setting	BCL6 Assessment	Comparator	Reproductive Outcomes Reported	Key Contribution
A. Comparative studies addressing the prognostic association between endometrial BCL6 expression and reproductive outcomes								
Almquist 2017 [15]	USA	Prospective cohort	Women with unexplained infertility undergoing IVF-ET; 69 transfers analyzed	Unexplained infertility before embryo transfer	LH-timed mid-luteal endometrial biopsy; IHC; HSCORE; abnormal > 1.4	Low/normal vs. high/abnormal BCL6	Clinical pregnancy; live birth per transfer	Lower BCL6 expression was associated with higher clinical pregnancy and live birth rates in women with unexplained infertility undergoing IVF.
Klimczak 2022 [17]	USA	Case–control	Women undergoing IVF with PGT-A followed by warmed single euploid embryo transfer; live birth group n = 27, no live birth group n = 23	Normal-responder IVF; single euploid FET	Endometrial biopsy; IHC; HSCORE; positivity cut-off > 1.4	BCL6-positive vs. BCL6-negative; live birth vs. no live birth	Live birth	No significant association was observed between BCL6 positivity and live birth in a selected euploid-transfer setting.
Strug 2025 [18]	USA	Prospective blinded observational cohort	IVF patients with subsequent euploid FET within 1 year; surgically confirmed endometriosis, unexplained infertility/RPL, and control groups; total n = 76	Euploid FET after fresh-cycle biopsy	Endometrial biopsy 5–7 days after oocyte retrieval; IHC for BCL6 and SIRT1; HSCORE > 1.4 considered positive	Correlation of BCL6/SIRT1 levels with later FET outcomes	Pregnancy outcomes after euploid FET, including live birth	BCL6 and SIRT1 levels were not associated with subsequent pregnancy outcomes in a euploid FET setting.
B. Comparative studies addressing treatment before embryo transfer in BCL6-positive women								
Likes 2019 [19]	USA/Brazil	Prospective cohort	Women with unexplained infertility and abnormal BCL6 before embryo transfer	Suspected endometriosis in infertility; ART/embryo transfer	Endometrial biopsy; IHC; abnormal BCL6 defined as HSCORE ≥ 1.4	Medical suppression or laparoscopy vs. no treatment among BCL6-positive women	Implantation; clinical pregnancy; live birth; miscarriage	Treated women had better reproductive outcomes than untreated controls.
Taggar 2025 [20]	USA	Retrospective chart review	IVF patients with positive BCL6 and an RPL/RIF-type infertility context	Post-biopsy intervention before transfer	Endometrial biopsy; standard IHC; positive BCL6 defined as HSCORE ≥ 1.4	Treated vs. untreated BCL6-positive women	Implantation; miscarriage; ongoing pregnancy	No significant differences were observed between treated and untreated BCL6-positive women.
C. Contextual studies included for narrative interpretation only								
Fox 2019 [13]	USA/Brazil	Case–control	Fertile controls, unexplained recurrent pregnancy loss, and unexplained infertility groups; total n = 110	uRPL and unexplained infertility	LH-timed mid-secretory endometrial biopsy; Western blot and IHC/HSCORE	Fertile controls vs. uRPL vs. UI	Not designed as a post-biopsy reproductive outcome study	Elevated BCL6 expression was observed in both uRPL and unexplained infertility groups.
Huang 2023 [21]	USA	Retrospective cohort	Infertility patients undergoing biopsy for BCL6 evaluation; n = 244	Frozen embryo transfer preparation protocols	Endometrial biopsy; HSCORE; overexpression > 1.4	Natural cycle vs. modified natural cycle vs. programmed cycle	No direct reproductive outcome comparison aligned with the review question	Uterine preparation protocol influenced measured BCL6 expression.
Huang 2025 [22]	USA	Retrospective clinical cohort plus pilot transcriptomic study	Patients undergoing concurrent ERA and ReceptivaDx; n = 172 in the clinical cohort	Endometrial receptivity testing in infertility	ReceptivaDx BCL6 HSCORE > 1.4 plus ERA and single-nucleus RNA sequencing	BCL6-positive vs. BCL6-negative in relation to ERA and receptivity markers	No direct reproductive outcome comparison aligned with the review question	BCL6 positivity was discordant with ERA-defined prereceptivity and related receptivity markers.
Lessey 2024 [33]	USA/Brazil	Mixed observational study with pilot randomized mechanistic component	Women with unexplained euploid embryo transfer failure; treatment and mechanistic substudies	UEETF; suspected endometriosis; suppression before transfer	Endometrial biopsy with BCL6 and SIRT1 testing	GnRH agonist or antagonist suppression vs. controls/OCP, depending on substudy	Cycle outcomes and inflammatory/epigenetic endpoints	Suggested mechanistic links between suppression of suspected endometriosis/BCL6-related pathology, inflammatory modulation, and possible clinical benefit.
Ekemen 2023 [31]	Turkey	Retrospective observational study	Women with unexplained infertility and recurrent IVF failure	UI/recurrent IVF failure	Endometrial biopsy with CD56, BCL6, and CD138 staining; digital pathology	Multimarker panel rather than isolated BCL6 comparator	Pregnancy rates after targeted therapy	Supported interpretation of BCL6 within combined immune-marker panels rather than as an isolated biomarker.
Evans-Hoeker 2016 [12]	USA/Brazil/Chile	Case–control series	Women with and without endometriosis, fertile controls, and unexplained infertility cohorts	Endometriosis, unexplained infertility, endometrial dysfunction	Prospectively collected samples; mRNA and IHC; ROC-derived HSCORE cut-off 1.4	Endometriosis/UI/fertile controls	Not a direct reproductive outcome study under the current framework	Demonstrated an association between elevated endometrial BCL6 and endometriosis/unexplained infertility and supported the HSCORE threshold of 1.4.
Nezhat 2020 [16]	USA	Retrospective cohort	IVF patients with unexplained infertility or unexplained recurrent pregnancy loss undergoing laparoscopy after positive BCL6 testing; n = 75	Suspected endometriosis before embryo transfer	Endometrial BCL6 overexpression testing	Positive BCL6 with surgical confirmation of endometriosis/inflammatory pelvic pathology	No direct treated-vs-untreated or high-vs-low reproductive comparison	Positive endometrial BCL6 showed high positive predictive value for endometriosis or other inflammatory pelvic pathology.
Squatrito 2022 [32]	Belgium	Comparative methodological observational study	Fertile and infertile women with or without endometriosis	Endometriosis-related infertility	Endometrial BCL6 quantified by HSCORE and digital image analysis	Fertile vs. infertile/endometriosis groups; analytical comparison of methods	No direct reproductive outcome comparison under the review question	Contributed to methodological standardization and reproducibility of BCL6 quantification, including digital image analysis.

Abbreviations: ART, assisted reproductive technology; BCL6, B-cell lymphoma 6; ET, embryo transfer; FET, frozen embryo transfer; GnRH, gonadotropin-releasing hormone; HSCORE, histologic score; IHC, immunohistochemistry; IVF, in vitro fertilization; OCP, oral contraceptive pill; PGT-A, preimplantation genetic testing for aneuploidy; RIF, recurrent implantation failure; RPL, recurrent pregnancy loss; UI, unexplained infertility; uRPL, unexplained recurrent pregnancy loss; UEETF, unexplained euploid embryo transfer failure. Note: Studies were grouped according to their contribution to the review questions: comparative studies addressing the prognostic association between endometrial BCL6 expression and reproductive outcomes; comparative studies addressing treatment before embryo transfer in BCL6-positive women; and contextual studies included for narrative interpretation only when they did not provide a comparator structure fully aligned with the main comparative questions or were primarily informative for biological, diagnostic, mechanistic, or methodological interpretation.

**Table 2:** Study-level risk-of-bias assessment of included studies.

Study	Selection Bias	Confounding	Exposure/Intervention Assessment	Outcome Assessment	Missing Data	Selective Reporting	Overall Risk
Almquist 2017 [15]	Moderate	Moderate	Low	Low	Low	Moderate	Moderate
Klimczak 2022 [17]	Moderate	Moderate	Moderate	Low	Low	Moderate	Moderate
Strug 2025 [18]	Moderate	Moderate	Low	Low	Moderate	Moderate	Moderate
Likes 2019 [19]	Moderate	Serious	Low	Low	Low	Moderate	Serious
Taggar 2025 [20]	Moderate	Serious	Moderate	Moderate	Moderate	Moderate	Serious
Fox 2019 [13]	Moderate	Moderate	Low	Moderate	Low	Moderate	Moderate
Huang 2023 [21]	Moderate	Moderate	Low	Low	Moderate	Moderate	Moderate
Huang 2025 [22]	Moderate	Moderate	Low	Moderate	Moderate	Moderate	Moderate
Lessey 2024 [33]	Moderate	Serious	Moderate	Moderate	Moderate	Moderate	Serious
Ekemen 2023 [31]	Moderate	Serious	Moderate	Moderate	Moderate	Moderate	Serious
Evans-Hoeker 2016 [12]	Moderate	Moderate	Low	Moderate	Low	Moderate	Moderate
Nezhat 2020 [16]	Moderate	Moderate	Moderate	Low	Moderate	Moderate	Moderate
Squatrito 2022 [32]	Moderate	Moderate	Low	Moderate	Low	Moderate	Moderate

Note: Risk-of-bias judgments were adapted pragmatically to the design and analytical role of each included study. For the five comparative studies central to the review questions, judgments reflect risks most relevant to prognostic or treatment-effect inference. For studies retained for contextual narrative interpretation only, judgments primarily reflect methodological credibility, selection issues, and interpretability within the review question. General study characteristics are presented in Table 1.

**Table 3:** Reproductive outcome data from studies evaluating high/abnormal versus low/normal endometrial BCL6 expression.

Study	Outcome	Low/Normal BCL6	High/Abnormal BCL6	Effect Estimate Reported by Study	Interpretation for This Review
Almquist 2017 [15]	Clinical pregnancy rate	11/17 (64.7%)	9/52 (17.3%)	RR for normal BCL6: 0.267 (95% CI 0.13–0.53); *p* = 0.0004	Lower/normal BCL6 expression was associated with markedly higher clinical pregnancy rates.
Almquist 2017 [15]	Live birth rate	10/17 (58.8%)	6/52 (11.5%)	RR for normal BCL6: 0.19 (95% CI 0.08–0.45); *p* = 0.0002	Lower/normal BCL6 expression was associated with markedly higher live birth rates.
Klimczak 2022 [17]	Live birth	20/35 (57.1%) in BCL6-negative group	7/15 (46.7%) in BCL6-positive group	OR for BCL6 positivity: 0.66 (95% CI 0.19–2.21); *p* = 0.49	No significant association between BCL6 positivity and live birth was observed.
Strug 2025 [18]	Live birth/pregnancy outcomes after euploid FET	Not directly interpretable in low-vs-high BCL6 format	Not directly interpretable in low-vs-high BCL6 format	Study reported no difference in BCL6 or SIRT1 levels between patients with and without live birth	Clinically informative for narrative synthesis, but not directly aligned with a simple low-versus-high BCL6 comparison.

Abbreviations: BCL6, B-cell lymphoma 6; CI, confidence interval; FET, frozen embryo transfer; OR, odds ratio; RR, risk ratio. Note: For Almquist 2017 [15], the published comparison was explicitly reported as low/normal versus high/abnormal BCL6. For Klimczak 2022 [17], the published data were reported as BCL6-positive versus BCL6-negative in women with versus without live birth, allowing derivation of outcome proportions for the two BCL6 groups. For Strug 2025 [18], the available data indicate no association between BCL6/SIRT1 levels and pregnancy outcomes, but the study does not appear to provide directly interpretable event counts in a simple high-versus-low BCL6 format in the material reviewed here.

**Table 4:** Reproductive outcome data from studies evaluating treated versus untreated BCL6-positive women before embryo transfer.

Study	Outcome	Treated BCL6-Positive	Untreated Comparator	Effect Estimate Reported by Study	Interpretation for This Review
Likes 2019 [19]	Implantation rate	GnRHa: 9/21 (42.9%); laparoscopy: 18/45 (40.0%)	12/103 (11.7%)	*p* < 0.0001	Implantation rate was higher after treatment than without treatment.
Likes 2019 [19]	Clinical pregnancy rate	GnRHa: 6/10 (60.0%); laparoscopy: 13/21 (61.9%)	8/54 (14.8%)	*p* = 0.002	Clinical pregnancy rate was higher in treated women.
Likes 2019 [19]	Live birth rate	GnRHa: 5/10 (50.0%); laparoscopy: 11/21 (52.4%); combined treated groups: 16/31 (51.6%)	4/54 (7.4%)	RR 6.9 (95% CI 2.5–18.9); absolute benefit 44.2%; NNT = 3	Strongest direct comparative signal supporting improved live birth after treatment in BCL6-positive women.
Likes 2019 [19]	Miscarriage rate	GnRHa: 1/6 (16.7%); laparoscopy: 2/13 (15.4%); combined treated groups: 3/19 (15.8%)	4/8 (50.0%)	Relative risk reduction 68.4% (95% CI—10.0 to 90.9); miscarriages reported as more common in controls	Treatment was associated with fewer miscarriages, although precision was limited.
Taggar 2025 [20]	Implantation rate	11/17 (65%)	3/5 (60%)	*p* = 0.85	No significant difference in implantation rate was observed between treated and untreated BCL6-positive women.
Taggar 2025 [20]	Ongoing pregnancy	9/17 (53%)	2/5 (40%)	*p* = 0.61	No significant difference in ongoing pregnancy was observed between groups.
Taggar 2025 [20]	Miscarriage rate	2/17 (12%)	1/5 (20%)	*p* = 0.64	No significant difference in miscarriage rate was observed between groups.

Abbreviations: BCL6, B-cell lymphoma 6; CI, confidence interval; GnRHa, gonadotropin-releasing hormone agonist; NNT, number needed to treat; RR, risk ratio. Note: Likes 2019 [19] provided the most complete direct comparative data for treated versus untreated BCL6-positive women before embryo transfer, including implantation, clinical pregnancy, live birth, and miscarriage outcomes. Taggar 2025 [20] reported implantation, ongoing pregnancy, and miscarriage outcomes for treated versus untreated BCL6-positive women, with no significant between-group differences. Angress 2020 [30] was retained only as supportive abstract-level evidence because its comparator was not a clearly defined untreated BCL6-positive group and adjusted comparative effect estimates were not available.

## Data Availability

The data analyzed in this study are derived from previously published studies and are available within the article and its Supplementary Materials.

## Supplementary Materials

The following supporting information can be downloaded at: https://www.mdpi.com/article/10.3390/diagnostics16111714/s1, Supplementary Table S1, detailed electronic search strategies; Supplementary Table S2, key sources of clinical and methodological heterogeneity across included studies; Supplementary Table S3, structured summary of findings across the prespecified clinical questions and key reproductive outcomes; Supplementary Table S4, GRADE assessment of certainty of evidence for the main reproductive outcomes; Supplementary Table S5, full-text articles excluded after eligibility assessment and principal reasons for exclusion; The PRISMA 2020 Checklist for the Systematic Review is provided as a separate supplementary file.

## Abbreviations

The following abbreviations are used in this manuscript:

ART	assisted reproductive technology
BCL6	B-cell lymphoma 6
BMI	body mass index
CI	confidence interval
CPR	clinical pregnancy rate
ET	embryo transfer
FET	frozen embryo transfer
GnRHa	gonadotropin-releasing hormone agonist
HSCORE	histologic score
IHC	immunohistochemistry
IVF	in vitro fertilization
LBR	live birth rate
LH	luteinizing hormone
OCP	oral contraceptive pill
OR	odds ratio
PGT-A	preimplantation genetic testing for aneuploidy
RIF	recurrent implantation failure
RPL	recurrent pregnancy loss
RR	risk ratio
SIRT1	sirtuin 1
UI	unexplained infertility
uRPL	unexplained recurrent pregnancy loss
UEETF	unexplained euploid embryo transfer failure
