# Supplementary material for: Endometrial BCL6 Expression and Reproductive Outcomes in Infertile Women: A Systematic Review
Source: Diagnostics (Basel). 2026 Jun 2;16(11):1714. doi: 10.3390/diagnostics16111714 (PMC13257124; doi:10.3390/diagnostics16111714)
Supplement: Supplementary file 1 [file diagnostics-16-01714-s001.zip › Supplementary Material.pdf]

Supplementary Material

Supplementary Table S1. Detailed electronic search strategies

Database	Search period	Database coverage	Detailed search strategy	Limits applied
MEDLINE (via PubMed)	15 December 2025 to 1 March 2026	From inception to 1 March 2026	("Infertility"[MeSH Terms] OR infertility[Title/Abstract] OR "unexplained infertility"[Title/Abstract] OR "recurrent implantation failure"[Title/Abstract] OR RIF[Title/Abstract] OR "recurrent pregnancy loss"[Title/Abstract] OR RPL[Title/Abstract] OR "in vitro fertilization"[Title/Abstract] OR IVF[Title/Abstract] OR "frozen embryo transfer"[Title/Abstract] OR FET[Title/Abstract]) AND ("Endometrium"[MeSH Terms] OR endometrium[Title/Abstract] OR endometrial[Title/Abstract] OR "endometrial biopsy"[Title/Abstract] OR biopsy[Title/Abstract]) AND ("BCL6"[Title/Abstract] OR "B-cell lymphoma 6"[Title/Abstract] OR "B-cell CLL/lymphoma 6"[Title/Abstract]) AND ("Embryo Transfer"[MeSH Terms] OR "embryo transfer"[Title/Abstract] OR implantation[Title/Abstract] OR "Pregnancy Outcome"[MeSH Terms] OR "pregnancy outcome"[Title/Abstract] OR "clinical pregnancy"[Title/Abstract] OR "live birth"[Title/Abstract] OR miscarriage[Title/Abstract])	No language restrictions; no date restrictions
Embase	15 December 2025 to 1 March 2026	From inception to 1 March 2026	('infertility'/exp OR infertility:ti,ab OR 'unexplained infertility':ti,ab OR 'recurrent implantation failure':ti,ab OR rif:ti,ab OR 'recurrent pregnancy loss':ti,ab OR rpl:ti,ab OR 'in vitro fertilization':ti,ab OR ivf:ti,ab OR 'frozen embryo transfer':ti,ab OR fet:ti,ab) AND ('endometrium'/exp OR endometrium:ti,ab OR endometrial:ti,ab OR 'endometrial biopsy':ti,ab OR biopsy:ti,ab) AND ('bcl6':ti,ab OR 'b-cell lymphoma 6':ti,ab OR 'b-cell cll/lymphoma 6':ti,ab) AND ('embryo transfer'/exp OR 'embryo transfer':ti,ab OR implantation:ti,ab OR 'pregnancy outcome'/exp OR 'pregnancy outcome':ti,ab OR 'clinical pregnancy':ti,ab OR 'live birth':ti,ab OR miscarriage:ti,ab)	No language restrictions; no date restrictions
Scopus	15 December 2025 to 1 March 2026	From inception to 1 March 2026	TITLE-ABS-KEY (infertility OR "unexplained infertility" OR "recurrent implantation failure" OR RIF OR "recurrent pregnancy loss" OR RPL OR "in vitro fertilization" OR IVF OR "frozen embryo transfer" OR FET) AND TITLE-ABS-KEY (endometrium OR endometrial OR "endometrial biopsy"	No language restrictions; no date restrictions

			OR biopsy) AND TITLE-ABS-KEY (BCL6 OR "B-cell lymphoma 6" OR "B-cell CLL/lymphoma 6") AND TITLE-ABS-KEY ("embryo transfer" OR implantation OR "pregnancy outcome" OR "clinical pregnancy" OR "live birth" OR miscarriage)	
Cochrane Central Register of Controlled Trials (CENTRAL)	15 December 2025 to 1 March 2026	From inception to 1 March 2026	(infertility OR "unexplained infertility" OR "recurrent implantation failure" OR RIF OR "recurrent pregnancy loss" OR RPL OR "in vitro fertilization" OR IVF OR "frozen embryo transfer" OR FET) AND (endometrium OR endometrial OR "endometrial biopsy" OR biopsy) AND (BCL6 OR "B-cell lymphoma 6" OR "B-cell CLL/lymphoma 6") AND ("embryo transfer" OR implantation OR "pregnancy outcome" OR "clinical pregnancy" OR "live birth" OR miscarriage)	No language restrictions; no date restrictions
Web of Science Core Collection: Science Citation Index Expanded	15 December 2025 to 1 March 2026	From inception to 1 March 2026	TS=(infertility OR "unexplained infertility" OR "recurrent implantation failure" OR RIF OR "recurrent pregnancy loss" OR RPL OR "in vitro fertilization" OR IVF OR "frozen embryo transfer" OR FET) AND TS=(endometrium OR endometrial OR "endometrial biopsy" OR biopsy) AND TS=(BCL6 OR "B-cell lymphoma 6" OR "B-cell CLL/lymphoma 6") AND TS=("embryo transfer" OR implantation OR "pregnancy outcome" OR "clinical pregnancy" OR "live birth" OR miscarriage)	No language restrictions; no date restrictions
Additional identification methods	15 December 2025 to 1 March 2026	Not applicable	Reference list screening of included studies and relevant reviews; forward citation searching; and contact with study authors or subject experts if required	Not applicable

Supplementary Table S2. Key sources of clinical and methodological heterogeneity across included studies

Study	Analytical grouping	Population / infertility phenotype	ART setting	Embryo context	Timing of endometrial biopsy	BCL6 assessment	Comparator structure	Main heterogeneity concern
Almquist 2017	Comparison 1: High/abnormal vs low/normal BCL6	Unexplained infertility	Fresh IVF-ET	Non-euploid / routine embryo transfer	LH-timed mid-luteal biopsy before IVF	IHC; HSCORE; abnormal >1.4	Low/normal vs high/abnormal BCL6	Unexplained infertility-only cohort; fresh-transfer setting

Klimczak 2022	Comparison 1: High/abnormal vs low/normal BCL6	Normal-responder IVF population	IVF with PGT-A followed by single euploid FET	Euploid embryos only	Mid-secretory biopsy during stimulation cycle	IHC; HSCORE >1.4	BCL6-positive vs BCL6-negative / live birth vs no live birth	Highly selected euploid-transfer population; reduced generalizability
Strug 2025	Comparison 1: High/abnormal vs low/normal BCL6	Endometriosis, unexplained infertility/RPL, and low-risk controls	IVF with later euploid FET	Euploid embryos only	Biopsy 5–7 days after oocyte retrieval in the fresh cycle	IHC for BCL6 and SIRT1; HSCORE >1.4	Correlation of marker levels with later pregnancy outcomes	Near-universal BCL6 positivity; mixed-risk groups; non-binary comparator structure
Likes 2019	Comparison 2: Treatment vs no treatment in BCL6-positive women	Unexplained infertility with abnormal BCL6 and suspected endometriosis	Fresh and frozen embryo transfer cycles	Mixed embryo context	LH-timed biopsy before treatment and embryo transfer	IHC; HSCORE ≥1.4	GnRHa or laparoscopy vs no treatment	Non-randomized treatment allocation; mixed treatment modalities; fresh and FET cycles combined
Taggar 2025	Comparison 2: Treatment vs no treatment in BCL6-positive women	RPL/RIF patients with positive BCL6	Post-biopsy embryo transfer cycle	Not uniformly restricted to euploid embryos	Luteal phase biopsy during infertility care	IHC; HSCORE ≥1.4	Treated vs untreated BCL6-positive women	Small sample size; retrospective design; heterogeneous interventions
Fox 2019	Contextual / qualitative synthesis only	Unexplained infertility and unexplained recurrent pregnancy loss	Not an ART outcome comparison study	Not applicable	Mid-secretory biopsy	Western blot and IHC/HSCORE	Fertile controls vs uRPL vs UI	Biological/phenotypic comparison rather than a treatment or prognostic outcome study
Huang 2023	Contextual / qualitative synthesis only	Infertility patients undergoing BCL6 evaluation	FET preparation setting	Transfer protocol-focused	Biopsy under different endometrial preparation methods	HSCORE; overexpression >1.4	Natural vs modified natural vs programmed cycle	BCL6 influenced by preparation protocol, complicating cross-study comparability
Huang 2025	Contextual / qualitative synthesis only	Infertility patients undergoing ERA and	Receptivity-testing context	Not primarily	Concurrent molecular/endometrial testing	ReceptivaDx BCL6 + ERA +	BCL6-positive vs BCL6-negative in relation to receptivity markers	Receptivity-focused rather than reproductive

		ReceptivaDx testing		outcome-based		transcriptomics		outcome-focused design
Lessey 2024	Contextual / qualitative synthesis only	Unexplained euploid embryo transfer failure	Euploid ET failure / suppression studies	Euploid embryo setting	Biopsy with mechanistic follow-up	BCL6 and SIRT1 testing	Mechanistic and treatment-oriented comparisons across substudies	Mixed clinical and mechanistic design; preliminary translational evidence
Ekemen 2023	Contextual / qualitative synthesis only	Unexplained infertility and recurrent IVF failure	IVF / infertility care	Mixed embryo context	Endometrial biopsy before targeted management	BCL6 within CD56/CD138 multimarker panel	Multimarker-guided management	BCL6 not isolated; panel-based interpretation reduces direct comparability
Evans-Hoeker 2016	Contextual / qualitative synthesis only	Endometriosis, unexplained infertility, and fertile controls	Not an embryo-transfer outcome study	Not applicable	Mid-secretory endometrial sampling	mRNA + IHC; ROC-derived cutoff 1.4	Endometriosis / UI / fertile controls	Foundational biological/diagnostic study rather than a reproductive outcome study
Nezhat 2020	Contextual / qualitative synthesis only	Unexplained infertility or unexplained RPL with positive BCL6	Pre-IVF / pre-ET diagnostic setting	Not primary focus	Endometrial BCL6 testing before laparoscopy	BCL6 overexpression testing	Positive BCL6 with laparoscopic confirmation	Diagnostic predictive-value design, not a comparative reproductive outcome study
Squatrito 2022	Contextual / qualitative synthesis only	Fertile and infertile women with or without endometriosis	Endometriosis-related infertility context	Not an ET outcome study	Endometrial sampling for analytical comparison	HSCORE and digital image analysis	Fertile vs infertile/endometriosis groups	Methodological standardization study, not an outcomes study

Abbreviations: ART, assisted reproductive technology; BCL6, B-cell lymphoma 6; ET, embryo transfer; FET, frozen embryo transfer; GnRHa, gonadotropin-releasing hormone agonist; HSCORE, histologic score; IHC, immunohistochemistry; IVF, in vitro fertilization; PGT-A, preimplantation genetic testing for aneuploidy; RIF, recurrent implantation failure; RPL, recurrent pregnancy loss; UI, unexplained infertility; uRPL, unexplained recurrent pregnancy loss.
Note: Heterogeneity across included studies was driven primarily by differences in clinical population, ART setting, embryo ploidy control, biopsy timing, comparator structure, and whether BCL6 was assessed as an isolated biomarker or within a broader diagnostic or multimarker framework.

Supplementary Table S3. Structured summary of findings across the prespecified clinical questions and key reproductive outcomes

Outcome	Clinical question	Evidence base among the 13 included studies	Direction of evidence	Main limitations	Overall interpretation
Clinical pregnancy	Is high/abnormal endometrial BCL6 expression associated with poorer reproductive outcomes than low/normal expression?	One direct comparative study (Almquist 2017) among the three studies grouped under Comparison 1	Favors low/normal BCL6	Very limited directly comparable evidence; lack of replication for clinical pregnancy across prognostic cohorts	Lower/normal BCL6 expression may be associated with higher clinical pregnancy rates, but the available evidence remains sparse
Live birth	Is high/abnormal endometrial BCL6 expression associated with poorer reproductive outcomes than low/normal expression?	Three studies in Comparison 1 (Almquist 2017, Klimczak 2022, Strug 2025)	Inconsistent	Marked heterogeneity in population selection, embryo ploidy control, biopsy timing, ART setting, and comparator structure	The adverse prognostic effect of high/abnormal BCL6 expression on live birth remains uncertain
Implantation	Does treatment before embryo transfer improve reproductive outcomes among BCL6-positive women?	Two studies in Comparison 2 (Likes 2019, Taggar 2025)	Inconsistent	Non-randomized treatment allocation, small sample sizes, and heterogeneous interventions	A treatment benefit for implantation is not consistently demonstrated across the included comparative studies
Clinical pregnancy	Does treatment before embryo transfer improve reproductive outcomes among BCL6-positive women?	One direct comparative study (Likes 2019) among the two studies grouped under Comparison 2	Favors treatment	Evidence is driven by a single prospective non-randomized cohort	Treatment before embryo transfer may improve clinical pregnancy in BCL6-positive women, but confirmatory comparative studies are needed
Live birth	Does treatment before embryo transfer improve reproductive outcomes among BCL6-positive women?	One direct comparative study (Likes 2019) among the two studies grouped under Comparison 2	Favors treatment, but limited	Evidence is dominated by one non-randomized cohort and has not been robustly replicated	Treatment before embryo transfer may improve live birth in selected BCL6-positive women, but certainty remains limited
Ongoing pregnancy / delivery	Does treatment before embryo transfer improve reproductive outcomes among BCL6-positive women?	One study (Taggar 2025) within Comparison 2	No clear difference	Small retrospective cohort with limited statistical power	Current evidence is insufficient to support a consistent benefit for ongoing pregnancy or delivery

Miscarriage	Does treatment before embryo transfer improve reproductive outcomes among BCL6-positive women?	Two studies in Comparison 2 (Likes 2019, Taggar 2025)	Inconsistent	Small numbers of events, partly retrospective evidence, and variable denominators	The effect of treatment on miscarriage remains uncertain
Biological and clinical interpretability of BCL6	Can endometrial BCL6 currently be considered a clinically robust stand-alone biomarker?	Eight studies included for contextual qualitative synthesis only (Fox 2019; Huang 2023; Huang 2025; Lessey 2024; Ekemen 2023; Evans-Hoeker 2016; Nezhat 2020; Squatrito 2022)	Mixed	Several included studies are diagnostic, mechanistic, methodological, or receptivity-focused rather than direct reproductive outcome studies	BCL6 appears clinically relevant, but the evidence from the 13 included studies does not support universal stand-alone use without clinical context

Abbreviations: BCL6, B-cell lymphoma 6.
Note: This table summarizes the direction, consistency, and clinical interpretability of the evidence across the 13 included studies in this review: three studies in Comparison 1, two studies in Comparison 2, and eight studies included for contextual qualitative synthesis only. It is intended as a structured narrative summary and does not represent a formal certainty-of-evidence assessment.

Supplementary Table S4. GRADE assessment of certainty of evidence for the main reproductive outcomes

Comparison / clinical question	Outcome	Studies (n)	Study design	Risk of bias	Inconsistency	Indirectness	Imprecision	Publication bias	Overall certainty of evidence	Plain-language interpretation
**Comparison 1:** Does high/abnormal endometrial BCL6 expression predict poorer reproductive outcomes than low/normal expression?	Clinical pregnancy	1 comparative study (Almquist 2017)	Observational cohort	Serious	Not serious	Serious	Very serious	Suspected	**Very low**	Abnormal/high BCL6 may be associated with lower clinical pregnancy in some infertility populations, but certainty is very low because evidence is based on a single observational study in a specific clinical setting.
**Comparison 1:** Does high/abnormal endometrial BCL6 expression predict poorer reproductive outcomes than low/normal expression?	Live birth	2 comparative studies contributing direct data (Almquist 2017; Klimczak 2022), with additional narrative support from Strug 2025	Observational cohort/case-control	Serious	Serious	Serious	Very serious	Suspected	**Very low**	The adverse prognostic effect of high/abnormal BCL6 expression on live birth remains uncertain. Findings differ across unexplained infertility and euploid embryo transfer settings, and pooled estimates are imprecise.
**Comparison 2:** Does treatment before embryo transfer improve reproductive outcomes among BCL6-positive women?	Implantation	2 comparative studies (Likes 2019; Taggar 2025)	Observational comparative studies	Serious	Serious	Serious	Very serious	Suspected	**Very low**	Treatment before embryo transfer may improve implantation in selected BCL6-positive women, but the evidence is inconsistent and highly uncertain.
**Comparison 2:** Does treatment before embryo transfer improve reproductive outcomes among BCL6-positive women?	Clinical pregnancy	1 direct comparative study (Likes 2019)	Observational cohort	Serious	Not serious	Serious	Very serious	Suspected	**Very low**	Treatment may improve clinical pregnancy in BCL6-positive women, but certainty is very low because the signal is driven by a single non-randomized study.

**Comparison 2:** Does treatment before embryo transfer improve reproductive outcomes among BCL6-positive women?	Live birth	1 direct comparative study (Likes 2019)	Observational cohort	Serious	Not serious	Serious	Very serious	Suspected	**Very low**	Treatment may improve live birth in selected BCL6-positive women, but certainty is very low because evidence is based on one non-randomized study and has not been robustly replicated.
**Comparison 2:** Does treatment before embryo transfer improve reproductive outcomes among BCL6-positive women?	Ongoing pregnancy / delivery	1 study (Taggar 2025)	Observational retrospective cohort	Serious	Not serious	Serious	Very serious	Suspected	**Very low**	Current evidence is insufficient to support a consistent benefit of treatment for ongoing pregnancy or delivery.
**Comparison 2:** Does treatment before embryo transfer improve reproductive outcomes among BCL6-positive women?	Miscarriage	2 comparative studies (Likes 2019; Taggar 2025)	Observational comparative studies	Serious	Serious	Serious	Very serious	Suspected	**Very low**	The effect of treatment on miscarriage remains uncertain because findings are inconsistent and based on small numbers of events.

Note: Certainty of evidence was assessed using the GRADE approach across the domains of risk of bias, inconsistency, indirectness, imprecision, and publication bias. Because all comparative evidence was observational, certainty was initially considered low and was further downgraded where appropriate. No upgrading was applied. Judgments were based on the small number of eligible comparative studies, non-randomized designs, clinically heterogeneous infertility populations, variation in embryo context, biopsy timing, BCL6 assessment strategy, comparator structure, and the imprecision of individual study estimates and the limited comparability of available outcome data. The additional conference abstract by Angress 2020 was not incorporated into the formal GRADE assessment because it did not meet the prespecified eligibility framework for primary comparative synthesis.

Abbreviations: BCL6, B-cell lymphoma 6.

Supplementary Table S5. Full-text articles excluded after eligibility assessment and principal reason for exclusion

Representative examples of full-text exclusions are presented below according to the principal exclusion categories applied during eligibility assessment.

Study	Study Type	Exclusion Category	Principal Reason for Exclusion
Mendeluk GR. *Increased Expression of BCL6 in Women with Implantation Failure* (2024)	Case series	No relevant reproductive outcomes	Included a very small number of patients and did not provide comparative reproductive outcome analysis aligned with the prespecified review questions.
Fox CW et al. *Unexplained Recurrent Pregnancy Loss and Unexplained Infertility: Twins in Disguise* (2019)	Observational cohort	Not directly relevant to the review question	Evaluated infertility phenotypes and BCL6-related findings without directly comparing reproductive outcomes according to the prespecified review framework.
Nezhat C et al. *BCL-6 Overexpression as a Predictor for Endometriosis in Patients Undergoing In Vitro Fertilization* (2020)	Diagnostic cohort study	Not directly relevant to the review question	Primarily focused on the diagnostic performance of BCL6 for endometriosis detection rather than comparative reproductive outcomes.
Nezhat C et al. *Can We Accurately Diagnose Endometriosis without a Diagnostic Laparoscopy?* (2022)	Narrative review	Review/non-original study	Review-based discussion of diagnostic approaches without original comparative clinical outcome data.
Squatrito M et al. *Comparison of Morphological and Digital-Assisted Analysis for BCL6 Endometrial Expression in Women with Endometriosis* (2022)	Methodological study	No relevant reproductive outcomes	Focused on methodological assessment of BCL6 quantification rather than reproductive outcome comparisons.
Gohari-Taban A et al. (2022)	Experimental study	Experimental/non-clinical design	Experimental/non-clinical design not eligible for inclusion in the comparative clinical synthesis.
Volovsky M, Seifer DB. *Current Status of Ovarian and Endometrial Biomarkers in Predicting ART Outcomes* (2024)	Narrative review	Review/non-original study	Review article without original comparative reproductive outcome data.
Koutalia N et al. *Role of Molecular Biomarkers in Endometriosis-Related Infertility* (2024)	Narrative review	Review/non-original study	Narrative biomarker review without eligible comparative clinical design.
Louwen F et al. *BCL6, a Key Oncogene, in the Placenta, Pre-Eclampsia and Endometriosis* (2022)	Review article	Review/non-original study	Mechanistic review article without original reproductive outcome comparisons.
Maziotis E et al. *Commercially Available Molecular Approaches to Evaluate Endometrial Receptivity* (2022)	Systematic review	Review/non-original study	Secondary evidence source without original comparative reproductive outcome data.
Garratt J, Rahmati M. *Assessing the Endometrium: An Update on Current and Potential Novel Biomarkers of Receptivity* (2023)	Review article	Review/non-original study	Narrative review focused on endometrial biomarkers and receptivity assessment.

Bakkensen JB et al. *Recent Advances and Current Perspectives on Endometrial Receptivity* (2021)	Review article	Review/non-original study	Review article without eligible primary comparative data.
Evans-Hoeker EA et al. *Endometrial BCL6 Overexpression in Eutopic Endometrium of Women with Endometriosis* (2016)	Mechanistic observational study	No relevant reproductive outcomes	Investigated biologic and molecular associations without comparative reproductive outcome analysis.
Huang DT et al. *B-Cell Lymphoma 6 Expression Significantly Differs by Uterine Preparation Method Used for Frozen Embryo Transfer* (2023)	Retrospective cohort study	Not directly relevant to the review question	Evaluated variation in BCL6 expression according to uterine preparation protocol rather than comparative reproductive outcomes.
Huang D et al. *A Positive ReceptivaDx Result for BCL6 Does Not Correlate with Abnormal ERA Results* (2025)	Retrospective cohort study	Not directly relevant to the review question	Focused on biomarker concordance and receptivity classification rather than reproductive outcome comparisons.
Ekemen S et al. *Endometrial Staining of CD56, BCL-6, and CD138 Improves Diagnosis and Clinical Pregnancy Outcomes* (2023)	Observational cohort study	Not directly relevant to the review question	Multimarker framework prevented isolated evaluation of BCL6-specific comparative effects.
Angress D. *Outcomes in Women with IVF Failure Who Tested Positive for BCL6 Using ReceptivaDx Testing* (2020)	Conference abstract	Not directly relevant to the review question	Conference abstract only, with insufficient methodological detail and incomplete outcome reporting for formal evidence synthesis.
Bui AH et al. *Evaluation of Endometrial Receptivity and Implantation Failure* (2022)	Review article	Review/non-original study	Narrative review without eligible primary comparative reproductive outcome data.
